# Therapeutic Exercise Parameters, Considerations and Recommendations for the Treatment of Non-Specific Low Back Pain: International DELPHI Study

**DOI:** 10.3390/jpm13101510

**Published:** 2023-10-19

**Authors:** Zacarías Sánchez Milá, Teresa Villa Muñoz, María del Rosario Ferreira Sánchez, Raúl Frutos Llanes, José Manuel Barragán Casas, David Rodríguez Sanz, Jorge Velázquez Saornil

**Affiliations:** 1 NEUMUSK Group, Facultad de Ciencias de la Salud, Universidad Católica de Ávila, 05005 Ávila, Spain; zacarias.sanchez@ucavila.es (Z.S.M.); mrosario.ferreira@ucavila.es (M.d.R.F.S.); jmanuel.barragan@ucavila.es (J.M.B.C.); 2 FisioSalud Ávila, 05002 Ávila, Spain; 3 Facultad de Enfermería, Fisioterapia y Podología, Universidad Complutense de Madrid, 28040 Madrid, Spain; davidrodriguezsanz@ucm.es

**Keywords:** Delphi study, low back pain, exercise, physiotherapy techniques, musculoskeletal disease

## Abstract

Background: Therapeutic exercise (TE) recommendations for non-specific low back pain (LBP) are meant to support therapy choices for people who suffer from this condition. The aim of this study was to reach an agreement on the definition and use of TE in the care of people with LBP. Methods: A Delphi study was carried out with a formal consensus procedure and sufficient scientific evidence, using an established methodology. Four rounds of anonymous questionnaires were administered to create useful suggestions and instructions in terms of the therapeutic activity for patients with LBP, and a group consensus conference. Results: A consensus was reached on most of the questions after 35 physiotherapists completed the questionnaires. Participants agreed that proper TE requires correct posture, body awareness, breathing, movement control, and instruction. Patients with LBP were advised to participate in supervised sessions twice a week for 30 to 60 min for a period of 3 to 6 months. Participants added that tailored evaluation and exercise prescription, monitoring, and functional integration of exercise, as well as using specific equipment, would benefit patients with LBP. Conclusions: TE recommendations for patients with LBP should be dosed and customized based on their personal psychological needs, level of fitness, and kinesiophobia.

## 1. Introduction

In order to be classified as low back pain (LBP), one must experience pain below the final set of ribs and above the buttock [1]. It is estimated that 80% of adults will experience LBP at some point in their lives [2,3,4]. LBP is a significant global public health issue. In recent years, it has been the leading cause of absence from work and medical rehabilitation needs. LBP is just one step behind mental health as a reason for early disability-based retirement [3,4,5,6]. In Germany, the disease management guidelines for non-specific LBP have been modified, stressing psychosocial workplace variables, early multidisciplinary therapy, and placing exercise ahead of bed rest [6]. Risk factors, such as certain regular postures that produce deviations, excess weight, and abdominal wall distension, may facilitate the development of non-specific LBP [3,7].

Only a small proportion of the population recognizes the pathological reason for their LBP, with 90% of cases being non-specific [2,3,4,5,6] and having an unknown medical cause [1].

In accordance with studies, the prevalence of non-specific LBP is 84% worldwide [1], increasing between the ages of 60 and 65, and decreasing steadily thereafter [6]. LBP is one of the main causes of disability worldwide, accounting for 54% of the increase in disability between 1990 and 2015 in low- and middle-income countries [8].

As some authors have stated, the higher prevalence of non-specific LBP in women than in men may be due to anatomical and functional differences. Women are smaller and have lower bone density, less muscular mass and weaker joints [9].

Patients frequently suffer from recurrent episodes of non-specific LBP. The annual incidence rate is higher in the third decade [2,3], ranging from 15% to 45%. Various medical and physiotherapeutic treatments are available, from the recommendation of analgesic measures to more conventional measures, such as passive therapies, including massage therapy and the application of electrotherapy for analgesic purposes. Nowadays, however, we are facing a paradigm change in which treatment is much more active on the patient’s side, and the term “hands off” has even been coined, referring to the previously explained concept of active therapy [7]. This new therapy is a therapeutic exercise (TE) tailored to the individual patient, in terms of intensity, the patient’s own pain and dosage. Physiotherapists are trained to treat various musculoskeletal complaints by means of TE and can prescribe such exercises [10].

It has been shown that patients with non-specific LBP experience less pain and disability when engaging in therapeutic activity [11,12,13]. According to recent searches, improvements are comparable regardless of the workout type [14,15,16]. When suggesting an exercise program for people with non-specific LBP, it is advisable to consider the logic behind TE approaches [14]. With this method, workout plans can be individually tailored for optimal effectiveness. Additionally, workouts that target postural control and trunk muscular stabilization may be advantageous for patients with non-specific LBP [12,13,14]. The evidence on the effectiveness of TE in patients with non-specific LBP, as claimed by some meta-analyses and systematic reviews, is, however, conflicting [14,15]. This result was caused by a lack of studies, varying methodological quality and small sample sizes [14]. As it would not be appropriate to combine the findings of these studies in a meta-analysis, the heterogeneity of the primary studies in terms of demographics, interventions, comparisons and outcome measures further restricts the robustness of the research findings.

To reach an agreement on the definition and use of TE to treat patients with LBP, a Delphi survey of a group of European physiotherapists was performed. The results of this study will help researchers to plan upcoming TE studies and to interpret previous research [16]. The Delphi survey’s study questions were:

- What are your qualifications in relation to people with non-specific LBP?
- What is the best TE design for patients with non-specific LBP in terms of guidelines, level of supervision and equipment?
- Which guidelines are used to ensure that TE is prescribed and progressed safely for patients with non-specific LBP?

## 2. Materials and Methods

An international group of traumatologists, orthopedic surgeons, fundamental scientists, physical activity and sports scientists, and surgeons with experience treating non-specific LBP attended a meeting. A formal consensus procedure was conducted with the use of a verified methodology (consisting of four rounds of questionnaires administered to a set of subject matter experts, conducted anonymously and not coinciding with each other) [17]. We analyzed the available research, convened a consensus group meeting to formulate recommendations and then organized a wider consultation meeting with an open invitation for final endorsement. With the help of local, national and international experts in non-specific LBP, we performed iterative consensus research (Delphi). The Delphi method is classified as one of the general foresight procedures aiming to obtain the consensus of a group of experts based on the analysis and reflection of a defined problem [17]. The members of this group were recruited using specific expressions of interest and invitations from experts and four rounds of anonymous questionnaires for the Delphi study. Rounds 1 and 2 consisted of the creation and ranking of a long list of potential traits, while in rounds 3 and 4 the participants were asked to agree on a set of preliminary criteria after being informed of the results of the previous rounds. Most of the participants (72%) were highly qualified and skillful European clinical volunteers in LBP management, reflecting different levels of clinical experience. Three levels of assurance were incorporated into the preliminary criteria from the earliest rounds: TE therapy is ideal, useless, or irrelevant. In the fourth round, consensus was reached with extremely high levels of agreement (>89%) amongst all levels of criteria and subcategories. Overall, 96% of the panelists agreed that the criteria should be adopted. The NEUMUSK research group of the Catholic University of Ávila, which designed the study, was in charge of supervising the correct methodological use of the Delphi Consensus at all times and was responsible for the storage and custody of the study results. In addition, it reviewed and approved the rules, followed by its experts in physiotherapy, along with other specialists in different fields such as physical activity and sports sciences, traumatology and orthopedics.

### Recruitment

**Figure 1:**
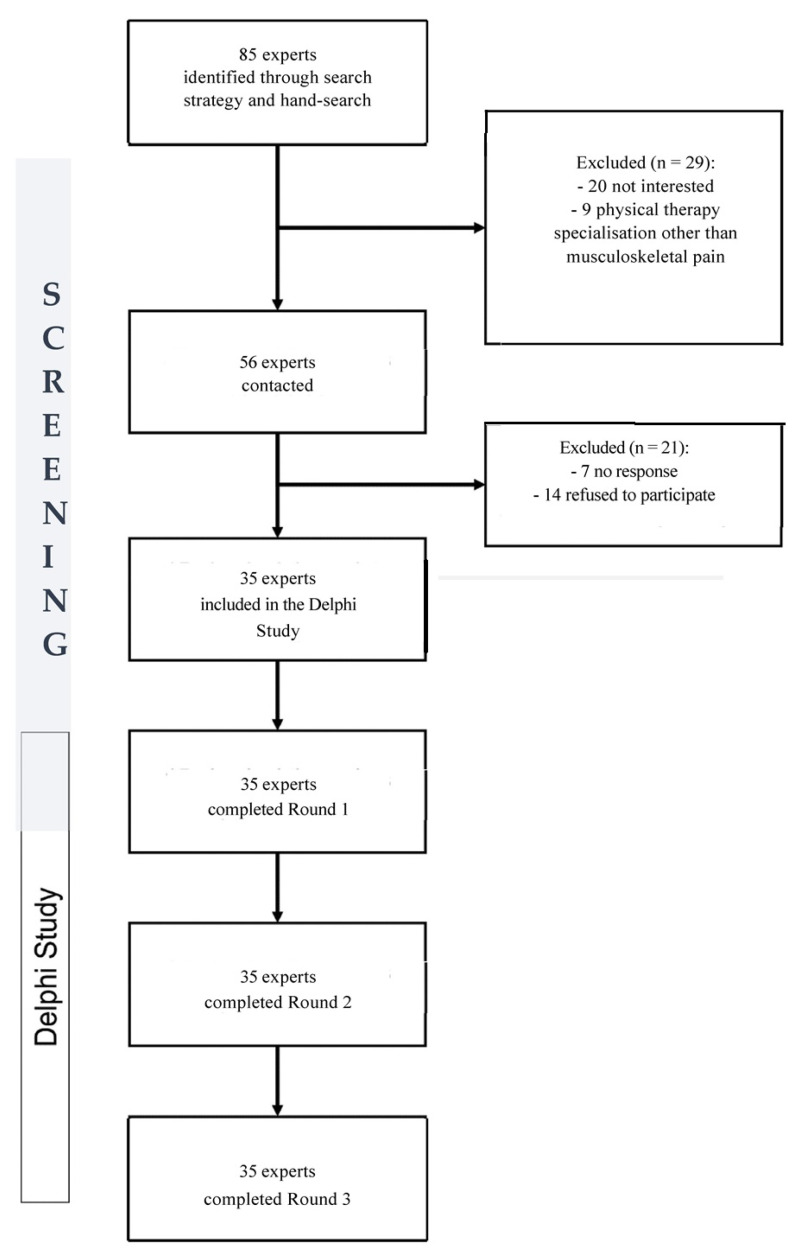
Expert recruitment diagram.

Participants were selected by means of purposive sampling, whereby a panel of “experts” were selected on the basis of their knowledge and experience of the topic, their availability and interest and skills to communicate. This selection method ensures that the results of the Delphi survey are based on informed opinions and that maximum participation rates are achieved. Snowballing techniques were also used to identify potential panel members. Snowballing techniques involve participants nominating or recommending others to participate in the study based on knowledge of the study’s inclusion criteria. The use of snowball recruitment techniques can increase both the size and diversity of the sample population. The recruitment process began with the principal investigator sending an email invitation to physiotherapists, physicians and physical activity and sport science experts who were likely to meet the selection criteria. This email included information about the research project and informed consent and screening forms. Participants were invited to contact the principal investigator by email or telephone to discuss the project. Participants were also encouraged to forward the project information to other interested professionals they thought might meet the selection criteria. Interested participants then emailed their completed screening and consent forms to the principal investigator. Once the screening and consent forms were received and checked, participants were formally included in the study. In the end, 35 participants made up the group of experts, as shown in Figure 1. The Delphi survey involved electronic questionnaires provided over the course of 5 months (May–September 2023). Participants were emailed electronic links to each questionnaire and received individual login details to complete their answers. Individual login details ensured the security of information and prevented duplicated responses. Participants were requested to complete each questionnaire within 2 weeks.

Responses to open-ended questions in the first questionnaire were summarized qualitatively using thematic analysis. Several researchers were involved in this process to ensure the validity and consistency of the approach. Themes identified from participant responses then were translated into statements about TE and people with LBP. These statements were utilized in the development of the next questionnaires.

Participants were requested to rank their level of agreement with a number of statements regarding TE in people with LBP using a 6-point Likert response scale (“strongly agree”, “agree”, “somewhat agree”, “somewhat disagree”, “disagree”, and “strongly disagree”). A 6-point Likert scale was selected because it has been shown to be valid, reliable, and suitable for use with educated individuals.

The Likert scale of responses was used to identify areas of consensus or non-consensus among the expert panel members. Prior to the commencement of this study, consensus was defined as when 70% to 100% of the panel members strongly agreed, agreed, or somewhat agreed (or strongly disagreed, disagreed, or somewhat disagreed) with an item. If the percentage of agreement or disagreement was less than 60%, however, it was concluded that a consensus had not been reached. Open-ended questions were also provided to ensure participants were able to express any further thoughts or opinions.

Below are the questionnaires and various questions sent to professionals to carry out the study using the Delphi Consensus methodology on the management of non-specific LBP pain using TE (questions 1 to 60 and tables from Tables A1–A21). 

## 3. Results

**Figure 2:**
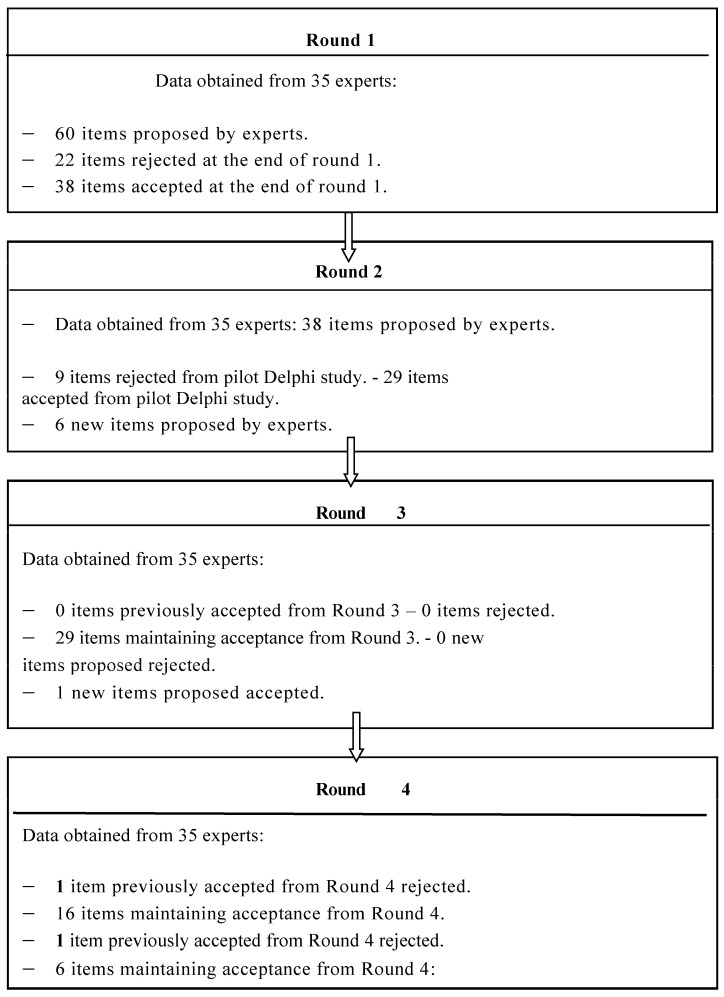
Consensus and four expert rounds.

The 35 specialists who participated in this study came to an agreement in the fourth round. TE is recommended as a course of treatment following a literature review and several multidisciplinary group sessions. The four expert rounds can be seen in Figure 2.

After four questionnaires, 91.7% (176/192) of the questions had consensus levels of agreement. However, 8.3% (16/192) of the questions could not be agreed upon. The components of consensus and non-consensus related to this study’s research topics are listed below.

What does “therapeutic exercise” mean in terms of those suffering from non-specific LBP?

From the questions regarding the definition of TE qualities, it was agreed that body awareness, breathing, control, education, individually adapted exercises, movement control and posture were identified as particularly significant elements of TE, specifically by 97.1% (33/34) of participants. 

Overall, 78.9% (15/19) of the critical elements of the TE protocol for patients with non-specific LBP were planned. The use of encouragement and feedback from the therapist, the functional integration of TE principles, the incorporation of home exercises, patient self-consciousness, and therapist reassessment were essential elements. Regarding the prescription of a specific number of exercises and the integration of resting and cooling activities, no agreement was reached.

Concerning the suitable parameters of TE and supervision for patients with non-specific LBP, an agreement was achieved within a range of values on 100% of the questions. Participants overwhelmingly agreed that supervised exercise sessions for patients with non-specific LBP should last between 30 and 60 min (100% agreement), should be performed twice a week (73.3% agreement), and should be completed within a period of 3 to 6 months (83.4% agreement).

These criteria were established, according to participant feedback, to make sure that clients remembered their exercises, used proper form, successfully corrected their motor patterns, strengthened their weak muscles, and accomplished their functional objectives. These guidelines also aimed to increase client satisfaction, motivation, and adherence within the existing constraints of availability and budget (100% agreement), as well as to enable the reduction, prevention, and self-management of symptoms and avoid frightened behavior.

One client per therapist was the suggested level of supervision by participants at the beginning of the program (80% agreement), and two to four clients per therapist after two weeks (100% agreement). Overall, 100% of participants believed that these degrees of supervision allowed for individualized exercise prescription, technique progression and monitoring, and ensured client self-care, pain and injury prevention, and a gradual decline in therapist dependence.

Furthermore, TE can supplement home activities, provide opportunities for growth, and offer customizable resistance. All questions relating to the customization of programs for people with non-specific LBP were agreed upon by the participants. Client goals, functional requirements, irritation, specific movement or activity anxieties, and body awareness are factors that should be given particular attention. A unanimous decision was obtained by all participants on all issues referring to the progression of exercise for those with non-specific LBP. The consensus among the participants was that the evolution of TE should have three main components: an increase in exercise complexity, a recreation involving a functional sport and the integration of exercise concepts.

The concept of prescribing TE to people with non-specific LBP was the subject of consensus on 94.7% (18/19) of the questions. Conducting an initial assessment, educating patients about the benefits of TE and chronic pain mechanisms, prescribing functionally relevant exercises in accordance with the client’s needs, ability, irritability, and pathology, supervising sessions, checking the effectiveness of the technique, encouraging breathing with movement, questioning belief systems to avoid fear, and routine reassessment of symptoms and functional outcomes were among the principles of great importance. Concerning the physical condition of patients, psychological state, and status in relation to kinesiophobia, no agreement was reached.

**Figure 3:**
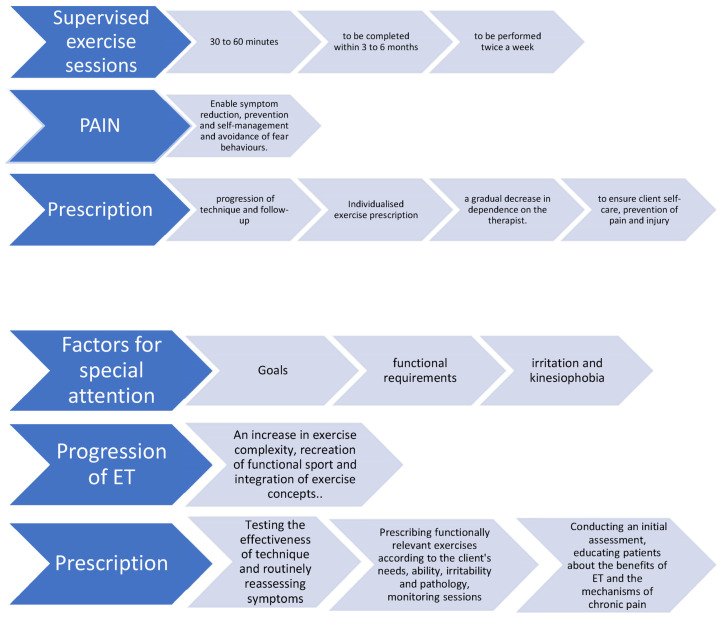
Agreements of the experts after the 4 rounds.

A summary of the decisions agreed upon by the experts after the four rounds can be seen in Figure 3.

## 4. Discussion

In this Delphi study, 35 health professionals agreed on most of the practical and definitional aspects of TE for people with non-specific LBP (Tabs 1). For 91.7% (176/192) of the items, consensus levels of agreement were attained after three rounds of questionnaires. The identification of TE features (1/34), must-have TE elements (4/19), essential equipment types (9/28) and their rationale for usage (1/11), and the basis of exercise prescription (1/19) were some of the points with non-agreement.

Participants concurred that all seven TE components—breathing, posture, flexibility, movement control, strength, core stability, and a mind–body connection—were appropriate for patients with non-specific LBP. These components were found in a recent systematic review of the literature [16]. The high median agreement reflected the importance placed on breathing, movement control, and posture. However, further investigation is needed to determine the relative importance of other distinctive qualities and vital elements.

Specific recommendations for the use of TE in the treatment of patients with non-specific LBP are provided by the consensus conclusions. The duration and frequency of TE sessions have been appropriate in view of these characteristics, but the length of exercise programs (i.e., 6–8 weeks) has often been inadequate [16,18]. Exercise trials for patients with non-specific LBP may find that the total number of sessions and hours of exercise are related to the effect size, so it may be important for future studies to make sure that TE interventions last between three and six months in order to achieve the best results [19].

The consensus conclusions also offer recommendations for the necessary tools and levels of supervision for applying TE to treat patients with non-specific LBP. The majority of TE trials for people with non-specific LBP have not used outside materials in their programs [20,21,22,23]. However, future research should examine the advantages of programs with and without the use of outside resources (use of materials such as elastic bands, rollers, etc.) considering the survey results. In future studies, grades of supervision should also be carefully considered because they could affect how well exercise works for people with non-specific LBP.

The guidelines for prescribing therapeutic exercise, which are similar to other exercise regimens that are successful in treating patients with non-specific LBP, were agreed upon by the participants. Participants, for instance, agreed that exercises should include stretching and strengthening and be individually designed and monitored [24,25,26]. Moreover, therapeutic activities should emphasize the coordination, strength, and endurance of the trunk muscles, respect clients’ treatment preferences (in the case of kinesiophobia) and incorporate cognitive behavioral therapy [27]. Additional clinical research is required to confirm the significance of further parts of the consensus related to individualization, prescription, and progression of workouts.

In published studies of participants with non-specific LBP, principles of therapeutic exercise, such as pelvic scale, concentration, and precision, were not discussed, indicating that they may not be significant according to our systematic analysis of the literature [15,16]. However, the principles of attention, accuracy, flow, pelvic scale, control, and breathing were taken into consideration while looking at consensus conclusions regarding the identification of TE features [27,28,29,30]. Although the CORE activation via the pelvic scale was the premise most frequently mentioned, high-intensity intervallic exercise for chronic pain should also be considered [31]. 

There are intrinsic limits to the Delphi method itself. Even if participants do not immediately contact each other, the iterative and anonymous group feedback process may persuade participants to agree. Bias among participants and researchers may result from this procedure. The results from Delphi surveys are only admissible as professional opinions and are ranked lower than primary studies in the hierarchy of evidence. A consensus of results does not necessarily imply that the group’s assessment is accurate. Therefore, these results need to be verified and put to the test in other clinical studies. 

Only 35 experts participated in this Delphi survey, which means that findings may be skewed, as only a proportion of physical therapists, physicians and physical activity and sports professionals experienced in the use of TE in people with LBP gave their opinion. Selection and response biases are likely to be present where physical therapists, physicians and physical activity and sports professionals who met the selection criteria were not invited to participate, did not agree to participate, or did not follow through in completing questionnaires.

## 5. Conclusions

The experts in the study concluded that TE approaches for patients with non-specific low back pain should be dosed and personalized according to the patient’s psychological needs, fitness level and kinesiophobia. In addition, they should be adapted to the level of pain of the patients, should be professionally supervised exercises lasting 30 to 60 min, twice a week and for at least 3 to 6 months. These results help us to understand how health professionals treat people with non-specific low back pain through TE. Future research on TE will benefit from this information, although it is important to evaluate the results in light of the limitations of the study.

## Data Availability

Not applicable.

## Appendix A

**Definition and Application of TE to Treat Non-specific Low Back Pain**
**Delphi Survey: Questionnaire**

The following questions (1–8) will help obtain consensus on the definition of TE and guidelines for the treatment of people with nonspecific low back pain (LBP). Please provide a response and rationale for each of your answers.

1. Describe the identifying characteristics of TE that are relevant to the physiotherapeutic treatment of people with LBP.
2. Describe principles that you use to guide safe and effective prescription of TE in people with LBP. Explain your rationale.
3. Describe principles that you use to guide safe and effective progression of TE in people with LBP.
4. What is the ideal length of TE sessions for people with LBP (in minutes)? Explain your rationale.
5. What is the ideal frequency of TE sessions for people with LBP (number of sessions/week)? Explain your rationale.
6. What is the ideal duration of TE programs for people with LBP (number of weeks)? Explain your rationale.
7. What is the ideal degree of physical therapist supervision during TE sessions for people with LBP (number of clients: physical therapist)? Explain your rationale.
8. List essential equipment (if any) required to conduct an ideal TE session for people with LBP. Explain your rationale.

The following questions ask for demographic information that will enable analysis of survey responses with respect to participant characteristics. Multiple-choice and short-answer questions will be used to gather this information.

9. What is your age (in years)?
10. What is your gender? Select appropriate answer.

Male □

Female □

11. Select your highest level of university qualification related to physical therapy.

Bachelor’s degree

Bachelor’s honors degree

Master’s degree (course work)

Master’s degree (research)

Doctorate (PhD)

Doctorate of clinical physical therapy

Entry-level doctoral degree

Other—please specify:

12. How many years have you been registered as a physical therapist, doctor or physical trainer?
13. Where do you usually teach TEto people with LBP?

Public hospital □

Private hospital □

Private physical therapy practice □

Gym or fitness center

Other—please specify:

14. In which country territory do you predominately practice physical therapy or your speciality?

Europe

South America

North America

Asia

Australia

15. Have you participated in any TE training courses?

Yes □

No □

16. Which TE training courses have you attended? You may select more than 1 option.
17. In a typical week, what percentage of clientele who you treat present with LBP?

5%

1%

15%

2%

25%

>25%

18. In a typical week, what percentage of clientele with LBP would you treat with TE?

0–25%

26–5%

51–75%

≥76%

19. The following features have been suggested as important in describing TE as it relates to people with non-specific low back pain (LBP). Using the scale provided, please rate your level of agreement as to the importance of these features.

**Table A1:** Level of agreement for describing TE in relation to people with non-specific LBP.

	Strongly Agree	Agree	Somewhat Agree	Somewhat Disagree	Disagree	Strongly Disagree
Body awareness						
Breathing						
Cognitive-behavioral therapy						
Concentration						
Control						
Coordination						
Core stability						
Direction preference						
Education						
Endurance						
Flexibility						
Flow						
Goal oriented						
Graded						
Holistic						
Individualized						
Low impact						
Kinesiophobia						
Muscle balance						
Movement control						
Posture						
Precision						
Proprioception						
Relaxation						
Self-paced						
Supervised						
Strength						
Structured						

20. Please list any additional features that you feel are important when describing TE in relation to people with LBP.
21. What does “therapeutic exercise” mean in terms of those suffering from non-specific LBP?
22. The following components have been suggested as important to include in TE programs for people with LBP. Using the scale provided, please rate your level of agreement as to the importance of these components.

**Table A2:** Level of agreement for inclusion as important components in TE programs for people with LBP.

	Strongly Agree	Agree	Somewhat Agree	Somewhat Disagree	Disagree	Strongly Disagree
Education regarding therapeutic technique						
Warm-up exercises						
Cool-down exercises						
Minimum of 5 different therapeutic exercises						
Maximum of 1 different therapeutic exercises						
Rest periods between exercises						
Stretching exercises						
Therapist feedback on client technique						
Reassessment by therapist						
Prescription of home exercises						
Functional integration of exercises						

23. Please list any additional components that you feel are important to include in TE programs for people with LBP.
24. The following factors have been suggested as important to consider when designing an individual exercise program for a person with LBP. Please rate your level of agreement as to the importance of these factors.

**Table A3:** Level of agreement to include factors as important to consider when designing an individual exercise program for a person with LBP.

	Strongly Agree	Agree	Somewhat Agree	Somewhat Disagree	Disagree	Strongly Disagree
Body awareness						
Cardiovascular fitness						
Non-specificity of symptoms						
Client availability						
Goals						
Client commitment level						
Flexibility						
Functional limitations						
Intensity of pain						
Irritability						
Movement control						
Muscle strength						
Pathology						
Posture						
Psychosocial factors						
Kinesiophobia						
Previous therapeutic experience						

25. Please list any additional factors you feel are important to consider when designing an individual exercise program for a person with LBP.
26. Please select the ideal TE sessions for the majority of people with LBP.

<3 min

3 min

6 min

≥6 min

10 min

20 min

30 min

45 min

27. Please select the ideal frequency of supervised TE sessions for the majority of people with LBP.

5 sessions/week

4 sessions/week

3 sessions/week

2 sessions/week

1 session/week

28. Please select the ideal duration of a TE program for the majority of people with LBP.

<4 weeks

4 weeks

6 weeks

8 weeks

12 weeks

6 months

12 months

≥12 months

29. The following rationales have been suggested to underpin recommendations for TE parameters (e.g., session length, frequency, and duration) for people with LBP. Please rate your level of agreement as to the accuracy of these rationales.

**Table A4:** Level of agreement to include ET parameters in patients with non-specific LBP.

	Strongly Agree	Agree	Somewhat Agree	Somewhat Disagree	Disagree	Strongly Disagree
Enhance client self-management						
Ensure client remembers exercises						
Ensure client uses correct technique						
Ensure relearning of motor patterns						
Ensure strength changes occur						
Ensure treatment effectiveness						
Prevent recurrence of pain or kinesiophobia						

30. Please list any additional rationale you would suggest to underlie recommendations for TE parameters for people with LBP.
31. Please select the ideal level of supervision of TE for the majority of people with LBP.

1 client to 1 physical therapist

2 clients to 1 physical therapist

3 clients to 1 physical therapist

4 clients to 1 physical therapist

5 clients to 1 physical therapist

>5 clients to 1 physical therapist

32. The following rationales have been suggested to underpin level of supervision required for people with LBP undertaking therapeutic exercise.

**Table A5:** Level of supervision required for people with LBP undertaking TE.

	Strongly Agree	Agree	Somewhat Agree	Somewhat Disagree	Disagree	Strongly Disagree
Enable individual prescription of exercises						
Enable timely progression of exercises						
Encourage self-management and self-monitoring						
Ensure correct technique is used						
Prevent pain and injury						

33. Please list any additional rationale that underpins the level of supervision required for people with LBP undertaking therapeutic exercise.
34. The following equipment has been suggested as important for people with LBP undertaking therapeutic exercise. Using the scale provided, please rate your level of agreement as to the importance of these features.

**Table A6:** Equipment for people with LBP undertaking TE.

	Strongly Agree	Agree	Somewhat Agree	Somewhat Disagree	Disagree	Strongly Disagree
Balance disk						
Fit balls						
Foam rollers						
Hand weights						
Ladder Barrel						
Magic Circle						
Mirror						
Pressure biofeedback pillow						
Prop balls						
Raised bench/step						
Real-time ultrasound						
Reformer						
Step Barrel/spine corrector						

35. Do you consider the following equipment to be important for people with LBP doing TE? Using the scale provided, please rate your level of agreement on the importance of these features.

**Table A7:** Materials that can be used to perform ET in people with non-specific LBP.

	Strongly Agree	Agree	Somewhat Agree	Somewhat Disagree	Disagree	Strongly Disagree
Resistance bands						
Trapeze table						
Vibration machine						
Others						

36. Please list any additional equipment you feel is important for people with LBP undertaking therapeutic exercise.
37. The following rationales have been suggested to underpin use of Therapeutic equipment in people with LBP.

**Table A8:** Use of therapeutic equipment in people with LBP.

	Strongly Agree	Agree	Somewhat Agree	Somewhat Disagree	Disagree	Strongly Disagree
Able to grade exercises according to ability						
Adjust level of resistance						
Enable progression of exercises						
Increase exercise variation						
Increase proprioceptive feedback						

38. Please list any additional rationale underpinning the use of therapeutic equipment in people with LBP.
39. The following principles have been suggested as important to consider when prescribing TE for people with LBP. Rate your level of agreement as to the importance of these principles.

**Table A9:** Level of agreement to prescribing TE for people with LBP.

	Strongly Agree	Agree	Somewhat Agree	Somewhat Disagree	Disagree	Strongly Disagree
Conduct an initial physical therapy assessment						
Consider client directional bias						
Consider client irritability						
Consider client pathology						
Educate regarding the purpose of therapeutic exercise						
Ensure exercises do not cause or increase pain						
Gradually increase difficulty of exercises						
Monitor the quality of exercise technique						
Prescribe functionally relevant exercises						
Provide individualized exercises according to needs and ability						
Regularly reassess symptoms and functional outcomes						
Supervise exercise sessions						
Start in neutral spine position						
Teach traditional therapeutic principles						
Use specialized therapeutic equipment						

40. Please list any other principles you feel are important to consider when prescribing TE for people with LBP.
41. The following rationales were suggested to underpin the principles of TE prescription in people with LBP. Please rate your level of agreement as to the importance of each rationale.

**Table A10:** Principles of TE prescription in people with LBP.

	Strongly Agree	Agree	Somewhat Agree	Somewhat Disagree	Disagree	Strongly Disagree
Correct maladaptive movement patterns						
Decrease fear of movement						
Encourage appropriate muscle activation						
Ensure exercises are progressive						
Ensure movement is controlled						
Ensure treatment outcomes are reached						
Improve functional ability						
Improve posture and alignment						
Prevent aggravation of symptoms						

42. Please list any other rationale for prescription of TE in people with LBP.
43. The following ideas for progression of TE have been suggested for people with LBP. Rate your level of agreement as to the accuracy of these ideas.

**Table A11:** Progression of TE suggested for people with LBP.

	Strongly Agree	Agree	Somewhat Agree	Somewhat Disagree	Disagree	Strongly Disagree
Increase in exercise load or resistance						
Increase in exercise repetitions						
Increase in exercise duration						
Increase in exercise complexity						
Movement outside of directional preference						
Incorporation of segmental spinal movement						
Addition of limb movement with activation of stabilizing muscles of the lumbar spine						
Coordination of breathing and core stability muscle activation						
Functional integration of exercise principles						

44. Please list any additional ideas for progression of therapeutic exercises that you feel are relevant for people with LBP.
45. The following features have been suggested to be important in describing TE as it relates to people with non-specific LBP. Using the scale provided, please rate your level of agreement as to the importance of these features.

**Table A12:** Important features to describe TE in relation to people with non-specific LBP.

	Strongly Agree	Agree	Somewhat Agree	Somewhat Disagree	Disagree	Strongly Disagree
Fatiguing						
Functional						
Measured						
Mindfulness						
Pain-free						
Specific exercise						

46. The following components have been suggested as important to include in TE programs for people with LBP. Using the scale provided, please rate your level of agreement as to the importance of these components.

**Table A13:** Important components to include in TE programs for people with LBP.

	Strongly Agree	Agree	Somewhat Agree	Somewhat Disagree	Disagree	Strongly Disagree
Balance exercises						
Client ability to contract deep stabilizing muscles of the back						
Client self-reflection/correction						
Cool-down exercises						
Encouragement/positive feedback						
Feedback/cues regarding technique						
Low load, high repetitions						
Minimum of 5 different therapeutic exercises						
Maximum of 1 different therapeutic exercises						
Rest periods between exercises						
Screening for pelvic-floor dysfunction						
Strengthening exercises						
Stretching exercises						
Use of equipment						
Warm-up exercises						

47. The following factors have been suggested as important to consider when designing an individual exercise program for a person with LBP. Please rate your level of agreement as to the importance of these factors.

**Table A14:** Important factors to consider when designing an individual exercise program for people with LBP.

	Strongly Agree	Agree	Somewhat Agree	Somewhat Disagree	Disagree	Strongly Disagree
Client financial capacity						
Client motivation						
Functional requirements/outcomes						
Medication						
Pain management						
Pain-relieving exercises						
Previous exercise/sport experience						
Previous treatment and effect						
Screening for pelvic-floor dysfunction						
Specific movement/activity fears						
Time of day						

48. Please select the ideal length of TE sessions for the majority of people with LBP.

3 min.

6 min.

15 min.

30 min.

45 min.

49. Please select the ideal frequency of supervised TE sessions for the majority of people with LBP.

2 sessions/week

3 sessions/week

5 sessions/week

50. Please select the ideal duration of a TE program for the majority of people with LBP.

6 weeks

8 weeks

12 weeks

6 months

12 months

>12 months

51. The following rationales have been suggested to underpin recommendations for TE parameters (e.g., session length, frequency, and duration) for people with LBP. Please rate your level of agreement as to the accuracy of these rationales.

**Table A15:** Rationale to support recommendations on TE parameters.

	Strongly Agree	Agree	Somewhat Agree	Somewhat Disagree	Disagree	Strongly Disagree
Flexible according to client availability and budget						
To address psychosocial factors and fear avoidance						
To allow time for rest between exercise						
To assist client with exercise routine						
To enhance client adherence						
To establish functional goals						
To increase client enjoyment						
To increase client motivation						

52. Please select the ideal level of supervision of TE for the majority of people with LBP at the start of their exercise program.

1 client to 1 physical therapist

2 clients to 1 physical therapist

3 clients to 1 physical therapist

4 clients to 1 physical therapist

53. Please select the ideal level of supervision of TE for the majority of people with LBP after 2 weeks of their exercise program.

1 client to 1 physical therapist

2 clients to 1 physical therapist

3 clients to 1 physical therapist

4 clients to 1 physical therapist

54. Please select the ideal level of supervision of TE for the majority of people with LBP after 4 weeks of their exercise program.

1 client to 1 physical therapist

2 clients to 1 physical therapist

3 clients to 1 physical therapist

4 clients to 1 physical therapist

55. Please select the ideal level of supervision of TE for the majority of people with LBP after 6 weeks of their exercise program.

1 client to 1 physical therapist

2 clients to 1 physical therapist

3 clients to 1 physical therapist

4 clients to 1 physical therapist

56. Please select the ideal level of supervision of TE for the majority of people with Non-specific low back pain after 12 weeks of their exercise program.

1 client to 1 physical therapist

2 clients to 1 physical therapist

3 clients to 1 physical therapist

4 clients to 1 physical therapist

57. The following rationales have been suggested to underpin level of supervision required for people with LBP undertaking TE.

**Table A16:** Rationale to support the level of supervision required in TE.

	Strongly Agree	Agree	Somewhat Agree	Somewhat Disagree	Disagree	Strongly Disagree
Decrease dependence on therapist						
Consider client’s previous experience						

58. The following equipment has been suggested as important for people with LBP undertaking TE. Using the scale provided, please rate your level of agreement as to the importance of these features.

**Table A17:** Important equipment for people with LBP doing TE.

	Strongly Agree	Agree	Somewhat Agree	Somewhat Disagree	Disagree	Strongly Disagree
Chi ball						
Educational books						
Exercise handouts						
Franklin ball						
Magic Circle						
Massage ball						
Mat						
Pillows						
Stabilizer						
Ladder Barrel						
Suspension trainer						
Towels						
Vibration machine						
Video analysis						
Balance board						

59. The following rationales have been suggested to underpin decisions to use Therapeutic equipment to treat people with LBP.

**Table A18:** Use of therapeutic equipment to treat people with LBP.

	Strongly Agree	Agree	Somewhat Agree	Somewhat Disagree	Disagree	Strongly Disagree
Ability to maintain neutral spine						
Closed-chain afferent input						
Cost of equipment						
Space required for equipment						
Need to complement home exercises						
Ensure functional relevance						

60. The following principles have been suggested as important to consider when prescribing TE for people with LBP. Rate your level of agreement as to the importance of these principles.

**Table A19:** Principles to consider when prescribing TE for people with LBP.

	Strongly Agree	Agree	Somewhat Agree	Somewhat Disagree	Disagree	Strongly Disagree
Breathing with movement						
Educate regarding Non-specific pain mechanisms						
Muscle balance						
Target fear-avoidance/belief systems						
Teach traditional Therapeutic principles						

61. The following rationale was suggested to underpin the principles of TE prescription in people with LBP. Please rate your level of agreement as to the importance of this rationale.

**Table A20:** Principles of TE prescription in people with LBP.

	Strongly Agree	Agree	Somewhat Agree	Somewhat Disagree	Disagree	Strongly Disagree
Decrease client’s dependence on therapist						

62. The following ideas for progression of TE have been suggested for people with LBP. Rate your level of agreement as to the accuracy of these ideas.

**Table A21:** Ideas for the progression of ET for people with LBP.

	Strongly Agree	Agree	Somewhat Agree	Somewhat Disagree	Disagree	Strongly Disagree
Decrease base of support						
Increase speed of exercise						
Replicate functional tasks/sport						
Progress toward feared movements						
Reduce supervision and feedback						
